# The Ambiguous Loss Inventory Plus (ALI+): Introduction of a Measure of Psychological Reactions to the Disappearance of a Loved One

**DOI:** 10.3390/ijerph20065117

**Published:** 2023-03-14

**Authors:** Hannah Comtesse, Clare Killikelly, Sophie M. C. Hengst, Lonneke I. M. Lenferink, Simone M. de la Rie, Paul A. Boelen, Geert E. Smid

**Affiliations:** 1 Clinical and Biological Psychology, Catholic University Eichstaett-Ingolstadt, 85072 Eichstaett, Germany; 2 Department of Psychiatry, University British Columbia, Vancouver, BC V6T 2A1, Canada; 3 ARQ National Psychotrauma Centre, 1112 XE Diemen, The Netherlands; 4 Department of Clinical Psychology, Utrecht University, 3584 CS Utrecht, The Netherlands; 5 Department of Psychology, Health & Technology, University of Twente, 7522 NB Enschede, The Netherlands; 6 Department of Clinical Psychology and Experimental Psychopathology, Faculty of Behavioral and Social Sciences, University of Groningen, 9747 AE Groningen, The Netherlands; 7 Department of Humanist Chaplaincy Studies, University of Humanistic Studies, 3512 HD Utrecht, The Netherlands

**Keywords:** missing persons, disappearance, ambiguous loss, loss, grief, stress

## Abstract

Background: The disappearance of a significant person is an ambiguous loss due to the persistent uncertainty about the whereabouts of the person. Measures specifically capturing the psychological consequences of ambiguous loss are lacking. Therefore, this study aimed to develop the Ambiguous Loss Inventory Plus (ALI+) and evaluated its suitability for use with relatives of missing persons. Methods: ALI+ items were generated based on established measures for prolonged grief symptoms and literature on psychological responses to ambiguous loss. Eight relatives of missing persons (three refugees, five non-refugees) and seven international experts on ambiguous loss rated all items in terms of understandability and relevance on a scale from 1 (not at all) to 5 (very well). Results: On average, the comprehensibility of the items was rated as high (all items ≥ 3.7). Likewise, all items were rated as relevant for the assessment of common responses to the disappearance of a loved one. Only minor changes were made to the wording of the items based on the experts’ feedback. Conclusions: These descriptive results indicate that the ALI+ seems to cover the intended concept, thus showing promising face and content validity. However, further psychometric evaluations of the ALI+ are needed.

## 1. Introduction

The disappearance of a loved person can be referred to as ‘ambiguous loss’ [1] due to the persistent uncertainty about the whereabouts of the missing person. Boss [2] differentiates two types of ambiguous loss: when a person is physically absent but kept psychologically present by the family (e.g., divorce, disappearance) and when a person is physically present but mentally absent (e.g., coma or dementia). We focus solely on disappearances as a specific subtype of ambiguous loss. Natural disasters, armed conflicts, displacement, or forced disappearance (i.e., forced abduction by state agents) [3] are common reasons for the disappearance of people. Worldwide, tens of thousands of people go missing every year [4], specifically in countries affected by armed conflicts, although reliable statistics are scarce. Research confirms that a significant number of refugees have experienced the disappearance of a loved one. For example, it has been estimated that almost 100,000 persons have gone missing in Syria since 2011 [5]. In a representative survey of Syrian refugees in Sweden, 60% of participants reported the loss or disappearance of loved persons [6]. In addition, 19% of a representative sample of refugees in Australia indicated the disappearance or murder of at least one family member [7]. People also go missing in Western countries unaffected by conflicts, although there, the occurrence of a disappearance is comparatively rare [8].

Several studies have found elevated prolonged grief disorder (PGD) symptom levels in conflict-affected and refugee groups with disappeared loved ones [9,10,11]. PGD is characterized by severe grief symptoms (e.g., intense yearning or emotional pain, avoidance of loss-related reminders) causing distress and impairments in daily life for an extended period after the death of a loved person [12]. In addition, research has identified violent and potentially traumatic losses as being among the strongest risk factors for the development of PGD [13]. Therefore, it is not surprising that persons with ambiguous loss have an increased risk of developing PGD symptoms, but also other stress-related symptoms such as posttraumatic stress disorder or depression [9,11]; for systematic reviews, see [14,15].

However, the disappearance of a loved person appears to have different psychological consequences than the death of a significant other due to the uncertainty about the fate of the missing person and, thus, about the permanence of separation [2]. Typical reactions to a disappearance include extensive searching activities, confusion about roles, interpersonal conflicts on how to cope with the disappearance, and distress due to a lack of mourning rituals [16]. In addition, persistent thinking about the missing person and the circumstances of the disappearance, and hope for survival and return have been further identified by research [10,17]. Such cognitive, emotional, and behavioral reactions may be considered normal ways of coping with a situation of prolonged uncertainty. However, uncertainty complicates meaning attribution to the ambiguous loss of a loved one, which may be further complicated by social, economic, juridical, and other factors [18]. Furthermore, catastrophizing of one’s own reactions, depressive avoidance [19], counterfactual thinking [20], intolerance of uncertainty [21], maladaptive affect regulation strategies [22], and moderate levels of hope [10] have been identified as correlates of PGD symptoms in relatives of missing persons.

To assess the psychological consequences of a disappearance in research and practice, valid instruments are needed. So far, these consequences have solely been assessed with instruments developed for the assessment of correlates of prolonged grief [19] and ad hoc questions [10] or interviews [23]. Moreover, previous studies used established measures not specifically designed to assess symptoms after an ambiguous loss. Studies on PGD symptoms, for example, used validated grief measures referenced for the study purpose of a disappeared instead of a deceased person while answering the questions [9,18]. The situation after an ambiguous loss may not enable one to grieve as there is no possibility for closure [2]. Thus, established PGD measures may not capture precisely the distress after a disappearance. In research with families after ambiguous loss (e.g., military families with a missing father/husband, families with hospitalized children), a Boundary Ambiguity Scale [24] has been frequently used; for an overview, see [25]. After an ambiguous loss, family members need to reevaluate the system’s rules and roles and may experience boundary ambiguity as a specific consequence, that is, not knowing who belongs to the system [24]. Boundary ambiguity has also been associated with more PGD symptoms in Syrian refugees with missing loved ones [11]. However, as knowledge of the consequences of ambiguous losses is growing and increasingly recognized in refugee samples [6,7,11], the monitoring of specific symptoms after the disappearance of a loved one needs reconsideration. No questionnaire has yet been specifically developed to measure grief-like and more general reactions to the disappearance of a loved one.

To overcome this, the present study aimed to develop a measure of the specific psychological consequences of the disappearance of a significant person, called the Ambiguous Loss Inventory-Plus (ALI+). For this, we compiled items to assess psychological reactions specific to ambiguous loss. In the second step, relatives of missing persons evaluated the items with regard to comprehensibility and relevance for reactions to ambiguous loss. In the third step, the items were evaluated by international experts on ambiguous loss.

## 2. Materials and Methods

### 2.1. Generation of ALI+ Items: Expert Consensus

We followed current recommendations for the development phase of scales for behavioral and health research by first generating items and then asking experts and members of the target population to evaluate the items [26].

In the first step of this study, a three-part structure was chosen for the questionnaire, and items were developed. Parts 1 and 2 were based on the structure of the Traumatic Grief Inventory-Self Report (TGI-SR) [27] to measure losses and grief-like reactions. The TGI-SR assesses grief symptoms in response to the currently most distressing loss after indicating for a list of persons whether one has been confronted with the death of the specific person (e.g., partner, child, other relative) and, if so, when and under which circumstances. Part 3 was developed to assess more general psychological reactions specific to ambiguous loss (for the complete questionnaire, see Appendix A).

In part 1 of the ALI+, “losses” are asked about, and the number and type of losses, including deceased and missing persons, are inventoried. Further loss-related information is collected, including information about the relationship to the deceased or missing person, loss circumstances, time since loss, and details about the currently most distressing disappearance. These questions were drawn from the TGI-SR [27] and the measurement of interpersonal losses in a study on PGD symptoms among refugees [28]. The differentiation between deceased and missing persons was chosen to ease modular diagnostic assessments of groups with possible ambiguous losses and other loss experiences. For example, studies with refugees have shown that some of the most frequently reported traumatic experiences are sudden and violent death [29] and the disappearance of significant persons [6]. When using the ALI+ and a participant indicates a missing person as well as the death of a loved person, one would proceed with the presentation of parts 2 and 3 of the ALI+ and subsequently present a questionnaire for the assessment of PGD symptoms.

The items for part 2 of the ALI+, “separation distress related to the disappearance”, were extracted from the Traumatic Grief Inventory-Self Report Plus (TGI-SR+) [30]. With this, we aimed to capture grief-like reactions after an ambiguous loss similar to PGD symptoms after the experience of a death. We selected TGI-SR+ items mapping onto the DSM-5-TR criteria for PGD [12]. In addition, TGI-SR+ items assessing blame and inability to experience positive feelings were used. From the International Prolonged Grief Disorder Scale (IPGDS) [31], two items assessing preoccupation with the loss circumstances and cultural appropriateness of one’s reaction were used to make sure that the items aligned with both DSM-5-TR [12] and ICD-11 [32] diagnostic criteria for PGD. The wording of the selected items was referenced to the disappeared person or circumstances of the disappearance (e.g., changed from “I found myself longing or yearning for the person who died” to “Longing or yearning for the person who disappeared”; see Table 1). The instruction for the ratings (“Below, several separation distress reactions are listed. Please indicate how often you have experienced each of these reactions due to the disappearance of the person named above in the past month.”) was based on the instructions in the TGI-SR+. Further, a prefix to all part 2 ALI+ items was chosen to clarify the reference period (“In the past month, how often have you experienced …”). Thus, the wording of the items drawn from the TGI-SR+ and IPGDS was changed accordingly (e.g., from “I felt emotionally numb” to “Feeling emotionally numb”), as displayed in Table 1.

The selection of items in part 3 of the ALI+, “general psychological reactions to the disappearance”, was based on a literature review and a consensus expert meeting among the authors. We screened the literature for psychological reactions specific to the disappearance of a loved person (e.g., hope for return [10]; or an urge to help the person [33]). For the formulation of items, we considered that such reactions might have been studied as immanent components, correlates, or consequences of the response to the disappearance and do not have to represent symptoms. After discussion among the authors, consensus was reached upon which reactions to include as items (see Table 2). The instruction for the ratings of part 3 items was “Please indicate how often in the past month you have experienced the reactions listed below due to the disappearance of the person.” As in part 2 of the ALI+, a prefix to all part 3 items was used to clarify the reference period further (“In the past month, how often have you experienced …”).

### 2.2. Participants for Expert Evaluation of ALI+ Items

To evaluate the ALI+ items generated in step 1, both relatives of missing persons as well as experts in the field were asked to provide feedback on the ALI+ items.

For the second step of this study, relatives of missing persons were invited for their feedback. Inclusion criteria were: (a) experience of the disappearance of a relative, (b) age 18 years or older, and (c) sufficient knowledge of English or Dutch language to be able to give feedback on the items. Participants were drawn from two sources: (a) patients with ambiguous loss who were in treatment at ARQ Centrum ’45 for trauma-related disorders were informed by staff about the study, and (b) persons with missing family members from the research network of LL were invited to take part in the study. Participants recruited at ARQ Centrum ’45 provided feedback in a face-to-face questionnaire-based interview format conducted by SH or HC (*n* = 3), while participants from the research network of LL gave their feedback in an online survey (*n* = 5). All participants provided written informed consent before commencing the study.

In the third step, experts on ambiguous loss from the social networks of the authors were asked to evaluate the ALI+ items. Experts had to have (a) a PhD and experience in conducting and publishing research on ambiguous loss or (b) they had to work in advocating for the needs of persons with ambiguous loss or in treating people with stress-related disorders after ambiguous loss. The experts completed an online survey for their feedback (*n* = 7).

Ethical approval for conducting this study was obtained by the IRB of the Catholic University Eichstaett-Ingolstadt (number: 055-2021). Data collection took place between September 2021 and January 2022.

### 2.3. Procedure

Relatives and experts were asked to read each ALI+ item carefully and answer two questions on 5-point scales (1 = not at all, 5 = very well). The first question inquired about the comprehensibility of the item (“To what extent do you understand what is meant by this item?”), while the second related to its relevance (“To what extent does this item capture common feelings and reactions with regard to the disappeared person?”). In addition, the experts were asked for each item whether they had a general comment.

It was predefined that adaptations to the wording of an item would be considered based on the experts’ feedback (see Table 1 and Table 2) or when an item had been rated with an average score of ≤3 by relatives or experts. The predefined criterion for removal of an item from the questionnaire was that the relevance of the item had to have been assessed with a mean value of ≤3 by relatives or experts.

### 2.4. Statistical Analysis

To examine comprehensibility and relevance of the ALI+ items, descriptive statistics were used. For each ALI+ item, we reported mean values, standard deviations, and score range as scored by relatives of missing persons and experts.

## 3. Results

### 3.1. Characteristics of Relatives of Missing Persons

Characteristics of the eight relatives of missing persons who evaluated the ALI+ items are shown in Table 3. They were, on average, 55.6 years old and had, on average, 15.1 years of formal education. The majority were male (63%) and from the Netherlands (63%). Three relatives fled as refugees to the Netherlands and were in psychological treatment for trauma-related disorders.

### 3.2. Ratings

The relatives’ ratings of the ALI+ items are displayed in Table 4. None of the relatives rated an item as insufficient in terms of suitability for feelings and reactions to an ambiguous loss (all mean values for relevance were ≥3.2). In part 2, the highest mean relevance value of 4.5 (*SD* = 1.1) was obtained for item 9 (functional impairment), while the lowest relevance value of 3.4 (*SD* = 1.1) was achieved for item 8 (life is unfulfilling or meaningless). In part 3, the highest mean relevance score of 4.7 (*SD* = 0.4) was achieved for item 1 (thoughts about circumstances of the disappearance) and the lowest score of 3.2 (*SD* = 1.5) for item 8 (absence of proper ceremonies or rituals). On average, the comprehensibility of the items was rated as ≥4.1 in part 2 and ≥3.7 in part 3.

### 3.3. Characteristics of Experts

As shown in Table 3, seven experts on ambiguous loss evaluated the ALI+ items. All experts had obtained a PhD, and six out of the seven experts worked mainly in research. The majority of experts resided in Germany (four out of seven), while four experts were from Australia, Finland, the United Kingdom, and the United States, respectively.

### 3.4. Ratings

Table 5 summarizes the expert’s rating of the comprehensibility and relevance of each ALI+ item. None of the experts rated an item as insufficient in terms of its relevance for common responses to an ambiguous loss (mean relevance scores were ≥3.8). The highest mean relevance value of 5.0 was obtained for part 2, item 10 (negative thoughts about oneself), and the lowest mean value of 3.8 (*SD* = 0.9) was reported for part 2, item 4 (avoidance of reminders). In part 3, the highest mean relevance value of 5.0 was achieved for items 1 (thoughts about circumstances of the disappearance), 3 (thoughts about the current whereabouts), 4 (hope), and 9 (being no longer able to bear the uncertainty). The lowest mean relevance values in part 3 were achieved for items 7 (*M* = 4.3, *SD* = 0.7; urge to help) and 14 (*M* = 4.3, *SD* = 0.7; negative attitudes from others). The comprehensibility of the ALI+ items was rated on average as ≥4.2 in part 2 and ≥4.1 in part 3.

Based on the experts’ general comments, only minor adaptations were made to 5 out of 17 items in part 2 and 6 out of 11 items in part 3 (see Table 1 and Table 2). These adaptations included changes to the sentence structure of part 2 items 6, 9, and 13 (e.g., from “intense blame on others” to “blaming others” for item 13) to better match the prefix to all ALI+ items (“In the past month, how often have you experienced …”). We also changed part 2 items 2 and 16 as the experts remarked that the word “grief” included in these items was not suitable for the separation distress experience of relatives of disappeared persons. The experts further remarked that part 3 items 1, 3, 7, and 8 should be formulated more precisely to make them more suitable for an assessment of reactions to an ambiguous loss; we changed the items accordingly. A further comment was made that the item on searching behavior (part 3 item 2) should be reformulated as actual searching might not always be possible, but the urge to do so is likely to be present. Finally, for part 3, item 11, comments were made that the item was too complicated and included two different aspects (feeling of betrayal and difficulty accepting that one may never know what has happened). Therefore, we split this item into two separate items (see Appendix A).

## 4. Discussion

The aim of this study was to develop a specific measure for assessing the psychological consequences of the disappearance of a significant person, the ALI+, and to evaluate the face and content validity of the items for persons with disappeared loved ones. For this, experts and relatives of missing persons evaluated all developed items with regard to comprehensibility and relevance for reactions to an ambiguous loss.

For the development of the ALI+, only minor adaptations to the wording of items in parts 2 and 3 were made in response to the experts’ comments. In particular, we excluded the word “grief” from the respective part 2 items. As the experts noted in their comments, the situation of a missing person may not enable one to grieve as the whereabouts are unknown, and thus, there is no possibility for closure [2]. In a study with relatives of missing persons in Mexico, the perceived pressure for closure was identified as a barrier to psychosocial care despite high support needs [33]. Hence, we were careful to refer to “separation distress” rather than “grief” throughout the ALI+. With regard to the comprehensibility and relevance of the ALI+ items, the experts and relatives of missing persons rated all items as understandable in terms of capturing the corresponding separation distress or general psychological reaction. They further evaluated all items in terms of relevance for measuring common responses to an ambiguous loss. These results support a promising face and content validity of the ALI+. Mean values for the comprehensibility and relevance of all items were relatively high as assessed by experts and relatives of missing persons (all values ≥ 3.2, rated on 5-point scales). Therefore, no item had to be removed based on our criterion for item exclusion (i.e., a mean relevance value of ≤3 for the respective item). However, for the two ALI+ parts evaluated, the average comprehensibility and relevance ratings of the relatives were descriptively lower than those of the experts. One possible explanation for this could be that the relatives of missing persons evaluated each item and how they would have answered the item with regard to their current personal situation during the feedback. Items describing reactions not applicable to their own current situation might have been assessed with lower scale scores. 

This study resulted in the development of ALI+ items. In doing so, we followed current recommendations for the development phase of scales for behavioral and health research [26] by asking experts and members of the target population to rate each item’s relevance for an evaluation of content validity (for example, using 5 to 7 experts has been recommended [26], see also [34]). Regarding the face validity of the ALI+, all relatives of missing persons stated that it is evident that specific reactions to the disappearance are covered by this questionnaire. This becomes clear through comments made to specific items by the relatives because they were able to see what reaction the item was intended to assess, even if this did not reflect their current state (e.g., “This was virtually a non-stop thought for the first couple of years [name of the person] was missing. Unless you’re busy/distracted by life/work, I think thinking about where they might be is a default position for those left behind.”, “I constantly hoped that he was still alive, but I think many people struggle with this hope vs. the likely reality.”). Although our reliance on the TGI-SR+ and current literature on the psychological consequences of a disappearance in developing the ALI+ might have strengthened its content validity [26], we cannot rule that using more rigorous procedures in developing the ALI+ (e.g., starting with a large pool of items, cognitive interviews with relatives during completion) would have increased its content validity. 

There are several implications for future research with regard to the next steps of scale development of the ALI+. A thorough psychometric evaluation of the ALI+ in terms of construct validity, particularly with regard to the dimensionality of the items in parts 2 and 3, should be performed in future studies. With regard to convergent and divergent validity, we would expect the items in part 3 to be more strongly related to a conceptually similar construct like boundary ambiguity [24,25] than to a dissimilar construct like quality of life. It remains to be seen whether the responses assessed in part 3 of the ALI+ are normal ways of coping with an unbearable situation, e.g., [33] or correlates of mental health problems [10,17]. We are currently undertaking a study of the evaluation of the questionnaire’s psychometric qualities in refugees, but future studies with more diverse samples (e.g., including relatives of missing persons from a non-refugee background) are needed. In particular, the impact of different types of disappearances across different groups (e.g., disappearances in the context of armed conflicts, disappearances on the flight from the home country, or disappearances outside of war contexts) on the validity of the ALI+ should be examined. Although screening instruments for the most common mental disorders have been validated for refugees [35], screenings for ambiguous losses in this particularly vulnerable group are lacking. Despite a pending empirical evaluation of the ALI+, we propose to provisionally compute total scores on subscales based on part 2 and 3 items by summing items from each part. Higher scores might tentatively indicate stronger grief-like reactions (part 2) or other specific reactions to the disappearance (part 3) that, in combination with results based on established measures, could point to more distress in dealing with the uncertainty and possibly a need for support in coping with the not-knowing. Thus, the modular structure of the ALI+’s loss assessment and different questionnaire parts are intended to facilitate the inclusion of the assessment of specific consequences of an ambiguous loss in medical examinations with relatives of missing persons and for traumatized persons seeking psychological treatment. 

Besides several strengths of this study, such as including relatives of missing persons with and without a refugee background, several limitations need to be acknowledged. First, all relatives of missing persons had received some sort of psychosocial support, and the relatives with a refugee background were currently in treatment for mental health problems. This might have limited variation within the group of relatives. The experts on ambiguous loss were recruited from the authors’ network, and thus, our results may not generalize to other international experts. Second, we started the development of the ALI+ items on the basis of TGI-SR+ items and specific reactions to the disappearance identified in the literature. We, therefore, cannot be certain to what extent the ALI+ represents the full range of psychological reactions of relatives of missing persons. Still, we were able to get a preliminary picture of the suitability of the ALI+ items, which was the goal for this phase (rather than selecting items from a larger item pool) with a low sample size of eight relatives and seven experts [26]. Moreover, a larger sample size might have been difficult to achieve as relatives of missing persons have been shown to be a rare and hard-to-reach population [8]. Third, an additional evaluation step of letting relatives of missing persons verbalize their thoughts while filling in the ALI+ was not undertaken [26]. Therefore, possible difficulties with interpreting or understanding the ALI+ items’ content during the completion of the questionnaire cannot be ruled out. Finally, this study only focused on the English and Dutch versions of the ALI+. It is unclear how far our results generalize to other language groups.

## 5. Conclusions

This study resulted in the ALI+, a measure of psychological reactions specific to the disappearance of a significant person, allowing to capture a neglected response pattern to a specific loss common in specific groups such as refugees. All ALI+ items were rated as comprehensible and relevant for the assessment of common responses to an ambiguous loss by experts and relatives of missing persons. These descriptive results indicate a promising face and content validity of the ALI+. Future studies evaluating the reliability and validity of the ALI+ in different samples of persons with disappeared significant others are needed.

## Figures and Tables

**Table 1:** Items for part 2 of the ALI+ (separation distress related to the disappearance).

TGI-SR+ Item	ALI+ Item Presented to Relatives and Experts for Feedback	Adapted ALI+ Item Based on Expert Rating
I had intrusive thoughts or images related to the person who died	1. Intrusive thoughts or images related to the person who disappeared	n/a
I experienced intense emotional pain, sadness, or pangs of grief	2. Intense emotional pain, sadness, or pangs of grief	Intense emotional pain and sorrow
I found myself longing or yearning for the person who died	3. Longing or yearning for the person who disappeared	n/a
I avoided places, objects, or thoughts that reminded me that the person I lost has died	4. Avoidance of places, objects, or thoughts that reminded you that the person disappeared	n/a
I felt bitterness or anger related to his/her death	5. Bitterness or anger related to his/her disappearance	n/a
I felt that that moving on (e.g., making new friends, pursuing new interests) was difficult for me	6. Moving on (e.g., making new friends, pursuing new interests) was difficult for you	Difficulty reengaging with everyday life activities (e.g., making new friends, pursuing new interests)
I felt emotionally numb	7. Feeling emotionally numb	n/a
I felt that life is unfulfilling or meaningless without him/her.	8. Life is unfulfilling or meaningless without him/her	n/a
I noticed significant reduction in social, occupational, or other important areas of functioning (e.g., domestic responsibilities) as a result of his/her death	9. A significant reduction in social, occupational, or domestic functioning because of his/her disappearance	Impairment in social, work, or domestic functioning because of his/her disappearance
I had negative thoughts about myself in relation to the loss (e.g., thoughts about self-blame) ^a^	10. Negative thoughts about yourself in relation to the disappearance (e.g., thoughts about self-blame)	n/a
I felt alone or detached from other individuals	11. Feeling alone or detached from other individuals	n/a
It felt unreal that he/she is dead	12. Feeling it is unreal that he/she disappeared	n/a
I put an intense blame on others because of his/her death ^a^	13. Intense blame on others because of his/her disappearance	Blaming others because of his/her disappearance
It felt as if a part of me has died along with the deceased	14. A part of you is gone along with the person who disappeared	n/a
I had difficulties experiencing positive feelings ^a^	15. Difficulties experiencing positive feelings	n/a
My grief would be considered worse (e.g., more intense, severe and/or of longer duration) than for others from my community or culture ^b^	16. Your grief is worse (e.g., more intense, severe and/or of longer duration) than for others from your community or culture	Your reaction to the disappearance is worse (e.g., more intense, severe and/or of longer duration) than for others in a similar situation from your community or culture
I am preoccupied with thoughts about the deceased or circumstances of the death ^b^	17. Preoccupation with thoughts or images related to the person or disappearance	n/a

Note: n/a = not applicable. ^a^ This Traumatic Grief Inventory-Self Report Plus item assesses a PGD symptom according to ICD-11 but not DSM-5-TR. ^b^ This item is included in the International Prolonged Grief Disorder Scale for the assessment of a PGD symptom according to ICD-11.

**Table 2:** Items for part 3 of the ALI+ (general psychological reactions to the disappearance).

ALI+ Item Presented to Relatives and Experts for Feedback	Adapted ALI+ Item Based on Expert Rating
1. Thinking about the circumstances under which he/she disappeared	Preoccupation with the circumstances under which he/she disappeared
2. Searching for him/her	The urge to search for him/her
3. Thinking about where he/she currently might be	Worry about where he/she currently might be
4. Hope that he/she is still alive	n/a
5. Feeling that you can only move on with your life if you know what happened to him/her	n/a
6. Disagreements with others on how to deal with his/her disappearance	n/a
7. The urge to help him/her	Distress because you cannot do more for him/her
8. Being upset by the idea that he/she may have died in the absence of proper ceremonies or rituals (e.g., funeral)	Distress because of the absence of proper ceremonies or rituals (e.g., funeral)
9. Feeling no longer able to bear the uncertainty of what happened to him/her	n/a
10. Avoiding talking about his/her disappearance because it upsets you too much	n/a
11. Feeling like betraying or dishonoring him/her when accepting that you might never know what happened to him/her	Feeling like betraying him/her when you move on with your life Difficulty accepting that you might never know what happened to him/her
12. Lack of emotional and/or practical support in dealing with his/her disappearance	n/a
13. Confusion about your current role in life because of his/her disappearance	n/a
14. Negative attitudes from other people because of his/her disappearance	n/a

Note: n/a = not applicable.

**Table 3:** Characteristics of relatives of missing persons (*n* = 8) and experts (*n* = 7).

Characteristic	Relatives of Missing Persons	Characteristic	Experts on Ambiguous Loss
Gender, % (*n*)		Main field of work, % (*n*)	
Female	37 (3)	Research	86 (6)
Male	63 (5)	Practice	14 (1)
Country of birth, % (*n*)		Country of residence, % (*n*)	
Afghanistan	12 (1)	Australia	14 (1)
Morocco	12 (1)	Finland	14 (1)
Netherlands	63 (5)	Germany	44 (3)
Sierra Leone	12 (1)	United Kingdom United States	14 (1) 14 (1)
Relationship to the disappeared person, % (*n*)			
Child	37 (3)		
Brother/sister	50 (4)		
Partner	13 (1)		
Age in years, *M* (*SD*)	55.6 (7.9) [range: 37–63]		
Years of education, *M* (*SD*)	15.1 (2.4) [range: 11–18]		

**Table 4:** Comprehensibility and relevance of ALI+ items based on ratings from relatives of missing persons (*n* = 8).

ALI+ Item in Part 2 ^a^	Comprehensibility	Relevance	ALI+ Item in Part 3	Comprehensibility	Relevance
Abbreviation (Item Number)	*M* (*SD*) [Range]	*M* (*SD*) [Range]	Abbreviation (Item Number)	*M* (*SD*) [Range]	*M* (*SD*) [Range]
Intrusive thoughts (1)	4.7 (0.4) [4–5]	4.2 (0.8) [3–5]	Preoccupation with the circumstances (1)	4.8 (0.3) [4–5]	4.7 (0.4) [4–5]
Emotional pain (2)	4.7 (0.4) [4–5]	4.2 (1.1) [2–5]	Urge to search (2)	4.8 (0.3) [4–5]	4.6 (0.7) [3–5]
Yearning (3)	5.0 (0.0) [5]	3.5 (1.4) [1–5]	Current whereabouts (3)	3.7 (1.3) [2–5]	4.4 (1.1) [2–5]
Avoidance (4)	4.7 (0.4) [4–5]	3.7 (1.3) [1–5]	Hope (4)	4.8 (0.3) [4–5]	4.3 (0.8) [3–5]
Bitterness (5)	4.8 (0.3) [4–5]	4.1 (1.3) [1–5]	Moving on if you know what happened (5)	4.7 (0.6) [3–5]	4.2 (1.3) [1–5]
Difficulty moving on (6)	4.7 (0.7) [3–5]	3.8 (1.4) [1–5]	Disagreements with others on how to deal (6)	4.3 (0.8) [3–5]	3.7 (1.4) [1–5]
Numbness (7)	4.8 (0.3) [4–5]	3.8 (1.3) [2–5]	Cannot do more for him/her (7)	4.2 (1.1) [2–5]	4.2 (1.3) [1–5]
Life is unfulfilling (8)	4.7 (0.7) [3–5]	3.4 (1.3) [2–5]	Absence of ceremonies or rituals (8)	3.9 (1.1) [2–5]	3.2 (1.5) [1–5]
Functional impairment (9)	4.5 (0.7) [3–5]	4.5 (1.1) [2–5]	Uncertainty (9)	4.8 (0.3) [4–5]	4.6 (0.7) [3–5]
Negative thoughts about yourself (10)	5.0 (0.0) [5]	4.3 (1.4) [1–5]	Not talking about what happened (10)	5.0 (0.0) [5]	4.0 (1.5) [1–5]
Loneliness (11)	5.0 (0.0) [5]	4.4 (1.1) [2–5]	Betraying and difficulty accepting (11)	4.2 (1.1) [2–5]	4.0 (1.1) [2–5]
Disappearance is unreal (12)	4.4 (1.1) [2–5]	4.4 (1.1) [2–5]	Lack of support (12)	4.1 (1.1) [3–5]	4.2 (0.9) [2–5]
Blame on others (13)	4.4 (1.1) [2–5]	3.7 (1.6) [1–5]	Confusion about current role (13)	4.2 (0.8) [3–5]	3.7 (1.5) [1–5]
A part is gone (14)	4.7 (0.4) [4–5]	3.8 (1.4) [1–5]	Negative attitudes from others (14)	4.2 (1.1) [2–5]	3.6 (1.5) [1–5]
Difficulty with positive feelings (15)	4.7 (0.7) [4–5]	4.1 (1.1) [2–5]			
Reaction is worse (16)	4.8 (0.3) [4–5]	4.4 (1.1) [4–5]			
Preoccupation (17)	4.1 (1.3) [2–5]	3.6 (1.5) [1–5]			

Note: Comprehensibility and relevance were assessed with 5-point scales (1 = not at all, 5 = very well). ^a^ ALI + part 2 items 3–17 were rated by *n* = 7 relatives.

**Table 5:** Comprehensibility and relevance of ALI+ items based on ratings from experts on ambiguous loss (*n* = 7).

ALI+ Item in Part 2	Comprehensibility	Relevance	ALI+ Item in Part 3	Comprehensibility	Relevance
Abbreviation (Item Number)	*M* (*SD*) [Range]	*M* (*SD*) [Range]	Abbreviation (Item Number)	*M* (*SD*) [Range]	*M* (*SD*) [Range]
Intrusive thoughts (1)	4.2 (0.7) [3–5]	4.5 (0.7) [3–5]	Preoccupation with the circumstances (1)	5.0 (0.0) [5]	5.0 (0.0) [5]
Emotional pain (2)	4.6 (0.7) [3–5]	4.3 (0.7) [3–5]	Urge to search (2)	4.1 (1.2) [2–5]	4.8 (0.4) [4–5]
Yearning (3)	4.8 (0.4) [4–5]	4.6 (0.7) [3–5]	Current whereabouts (3)	5.0 (0.0) [5]	5.0 (0.0) [5]
Avoidance (4)	5.0 (0.0) [5]	3.8 (0.9) [3–5]	Hope (4)	5.0 (0.0) [5]	5.0 (0.0) [5]
Bitterness (5)	4.8 (0.4) [4–5]	4.0 (1.4) [1–5]	Moving on if you know what happened (5)	4.6 (0.5) [4–5]	4.6 (0.5) [4–5]
Difficulty moving on (6)	4.5 (0.7) [3–5]	4.1 (0.9) [3–5]	Disagreements with others on how to deal (6)	4.6 (0.7) [3–5]	4.8 (0.4) [4–5]
Numbness (7)	4.3 (0.7) [3–5]	4.3 (0.5) [4–5]	Cannot do more for him/her (7)	4.5 (1.1) [2–5]	4.3 (0.7) [3–5]
Life is unfulfilling (8)	5.0 (0.0) [5]	4.3 (0.7) [3–5]	Absence of ceremonies or rituals (8)	4.4 (1.2) [2–5]	4.4 (0.8) [3–5]
Functional impairment (9)	4.5 (0.9) [3–5]	4.3 (0.9) [3–5]	Uncertainty (9)	4.8 (0.4) [4–5]	5.0 (0.0) [5]
Negative thoughts about yourself (10)	5.0 (0.0) [5]	5.0 (0.0) [5]	Not talking about what happened (10)	5.0 (0.0) [5]	4.8 (0.4) [4–5]
Loneliness (11)	5.0 (0.0) [5]	4.8 (0.4) [4–5]	Betraying and difficulty accepting (11)	4.6 (0.7) [3–5]	4.5 (0.7) [3–5]
Disappearance is unreal (12)	4.6 (0.5) [4–5]	4.8 (0.4) [4–5]	Lack of support (12)	4.6 (0.5) [4–5]	4.5 (0.5) [4–5]
Blame on others (13)	4.9 (0.7) [3–5]	4.6 (0.7) [3–5]	Confusion about current role (13)	4.6 (0.5) [4–5]	4.8 (0.4) [4–5]
A part is gone (14)	4.8 (0.4) [4–5]	4.1 (0.7) [3–5]	Negative attitudes from others (14)	4.3 (0.5) [4–5]	4.3 (0.7) [3–5]
Difficulty with positive feelings (15)	5.0 (0.0) [5]	4.8 (0.4) [4–5]			
Reaction is worse (16)	4.2 (0.9) [3–5]	4.8 (0.4) [4–5]			
Preoccupation (17)	5.0 (0.0) [5]	4.8 (0.4) [4–5]			

Note: Comprehensibility and relevance were assessed with 5-point scales (1 = not at all, 5 = very well).

## Data Availability

Not applicable.

## Appendix A

### Appendix A.1. Ambiguous Loss Inventory Plus (ALI+)

This questionnaire contains three parts. In Part 1, asks you about the loss of loved ones due to death or disappearance. Part 2 asks you about separation distress reactions you may experience due to the most distressing disappearance of a loved one. In Part 3, asks you about general psychological reactions you may experience due to the most distressing disappearance of a loved one.

#### Appendix A.1.1. Part 1: Losses

In this part you are asked to: (1) Indicate whether or not you have experienced the death or disappearance of the person mentioned. (2) Write down the date that each person died or disappeared. (3) If the person died, please indicate if the person died due to violent causes (by which we mean death due to homicide, suicide, or some forcible cause).

**(1) I have been confronted with the death or disappearance of:**			**(2) Date of death or disappearance:**	**(3) Death was due to violent causes:**
	**Deceased**	**Disappeared**		**Yes**
Partner 1	☐	☐		☐
Partner 2	☐	☐		☐
Child 1	☐	☐		☐
Child 2	☐	☐		☐
Child 3	☐	☐		☐
Father	☐	☐		☐
Mother	☐	☐		☐
Brother 1	☐	☐		☐
Brother 2	☐	☐		☐
Brother 3	☐	☐		☐
Sister 1	☐	☐		☐
Sister 2	☐	☐		☐
Sister 3	☐	☐		☐
Friend/ acquaintance 1, namely…	☐	☐		☐
Friend/ acquaintance 2, namely…	☐	☐		☐
Friend/ acquaintance 3, namely…	☐	☐		☐
Other relative 1, namely …	☐	☐		☐
Other relative 1, namely …	☐	☐		☐
Other relative 1, namely …	☐	☐		☐

From all the persons who disappeared, as listed in Part 1, please select one person whose disappearance is currently most often on your mind or causing you the most distress. Write down the name of this person below and answer the following questions.

*The missing person that is currently most often on my mind is:*

__________________________________________________________________

What gender is the person?

☐ female ☐ male ☐ other

How old was the person when he/she disappeared?

___________________________ years old

What were the circumstances of the person’s disappearance?

__________________________________________________________________

__________________________________________________________________

When did the person disappear?

☐ less than 6 months ago ☐ 6 to 12 months ago ☐ 1 to 5 years ago

☐ 5 to 10 years ago ☐ 10 to 20 years ago ☐ more than 20 years ago

#### Appendix A.1.2. Part 2: Separation Distress Related to the Disappearance

Below, several separation distress reactions are listed. Please indicate how often you have experienced each of these reactions due to the disappearance of the person named above in the past month.

	**In the past month, how often have you experienced …**	**Not at all** **(1)**	**Rarely** **(2)**	**Sometimes** **(3)**	**Often** **(4)**	**Always** **(5)**
1	intrusive thoughts or images related to the person who disappeared	1	2	3	4	5
2	intense emotional pain and sorrow	1	2	3	4	5
3	longing or yearning for the person who disappeared	1	2	3	4	5
4	avoidance of places, objects, or thoughts that reminded you that the person disappeared	1	2	3	4	5
5	bitterness or anger related to his/her disappearance	1	2	3	4	5
6	difficulty reengaging with everyday life activities (e.g., making new friends, pursuing new interests)	1	2	3	4	5
7	feeling emotionally numb	1	2	3	4	5
8	life is unfulfilling or meaningless without him/her	1	2	3	4	5
9	impairment in social, work, or domestic functioning because of his/her disappearance	1	2	3	4	5
10	negative thoughts about yourself in relation to the disappearance (e.g., thoughts about self-blame)	1	2	3	4	5
11	feeling alone or detached from other individuals	1	2	3	4	5
12	feeling it is unreal that he/she disappeared	1	2	3	4	5
13	blaming others because of his/her disappearance	1	2	3	4	5
14	a part of you is gone along with the person who disappeared	1	2	3	4	5
15	difficulties experiencing positive feelings	1	2	3	4	5
16	your reaction to the disappearance is worse (e.g., more intense, severe and/or of longer duration) than for others in a similar situation from your community or culture	1	2	3	4	5
17	preoccupation with thoughts or images related to the person or disappearance	1	2	3	4	5

#### Appendix A.1.3. Part 3: General Psychological Reactions to the Disappearance

Please indicate how often in the past month you have experienced the reactions listed below due to the disappearance of the person.

	**In the past month, how often have you experienced …**	**Not at all** **(1)**	**Rarely** **(2)**	**Sometimes** **(3)**	**Often** **(4)**	**Always** **(5)**
1	preoccupation with the circumstances under which he/she disappeared	1	2	3	4	5
2	the urge to search for him/her	1	2	3	4	5
3	worry about where he/she currently might be	1	2	3	4	5
4	hope that he/she is still alive	1	2	3	4	5
5	feeling that you can only move on with your life if you know what happened to him/her	1	2	3	4	5
6	disagreements with others on how to deal with his/her disappearance	1	2	3	4	5
7	distress because you cannot do more for him/her	1	2	3	4	5
8	distress because of the absence of proper ceremonies or rituals (e.g., funeral)	1	2	3	4	5
9	feeling no longer able to bear the uncertainty of what happened to him/her	1	2	3	4	5
10	avoiding talking about his/her disappearance because it upsets you too much	1	2	3	4	5
11	feeling like betraying him/her when you move on with your life	1	2	3	4	5
12	difficulty accepting that you might never know what happened to him/her	1	2	3	4	5
13	lack of emotional and/or practical support in dealing with his/her disappearance	1	2	3	4	5
14	confusion about your current role in life because of his/her disappearance	1	2	3	4	5
15	negative attitudes from other people because of his/her disappearance	1	2	3	4	5
